# Carbon-Based Stimuli-Responsive Nanomaterials: Classification and Application

**DOI:** 10.34133/cbsystems.0022

**Published:** 2023-04-11

**Authors:** Chen Zhao, Jun Kang, Yuwen Li, Yan Wang, Xiaoying Tang, Zhenqi Jiang

**Affiliations:** School of Life Science, School of Medical Technology, Analysis & Testing Center, Beijing Institute of Technology, Beijing 100081, China.

## Abstract

Carbon-based nanomaterials, including carbon nanotubes, carbon nanospheres, and carbon nanofibers, are becoming a research hotspot due to their unique structure and good mechanical, thermal, electrical, optical, and chemical properties. With the development of material synthesis technology, they can be functionalized and used in various fields such as energy, environment, and biomedicine. In particular, stimuli-responsive carbon-based nanomaterials have stood out in recent years because of their *smart* behavior. Researchers have applied carbon-based nanomaterials to different disease treatments based on their stimulus-response properties. In this paper, based on stimuli-responsive carbon-based nanomaterials’ morphology, we categorize them into carbon nanotubes, carbon nanospheres, and carbon nanofibers according to their morphology. Then, their applications in probes, bioimaging, tumor therapy, and other fields are discussed. Finally, we address the advantages and disadvantages of carbon-based stimuli-responsive nanomaterials and discuss their future perspective.

## Introduction

With the increasing demand for carbon-based nanomaterials in medical, industrial, and environmental fields, there has been a marked increase in the synthesis methods and applications of novel carbon-based nanomaterials in recent decades. In practical applications, carbon-based nanomaterials have attracted much attention because of their wide distribution, low synthesis cost, and simple synthesis method. There are many ways to classify carbon-based nanomaterials based on their great variation in size, shape, and composition [1]. Among them, the most common classification method is based on the microstructure and physicochemical properties of the materials. Carbon-based nanomaterials are usually classified into carbon quantum dots (CQDs), fullerenes, carbon nanotubes, graphene and its derivatives, nanodiamond, and graphene oxide based on their physicochemical properties. Among them, carbon-based stimuli-responsive materials are sensitive and can significantly alter their behavior. They have high chemical stability and show great application potential. Carbon-based stimuli-responsive materials can be adjusted by surface functionalization. Under various stimuli, including magnetic field, temperature, pH, light, and humidity, the hydrogen bond changes in the structure of carbon-based stimuli-responsive materials and functional groups interact, which can improve their conductivity as well as other physical and chemical properties [2]. In addition, carbon-based stimuli-responsive materials also allow for chemical modification, which overcomes the problem of genetic compatibility in cancer therapy and makes them a better drug carrier [3]. Carbon-based stimuli-responsive nanomaterials can be used as smart materials with dynamically tunable physicochemical properties in response to changes in internal or external environmental stimuli. Their diverse combinations of nanostructures and molecular designs, as well as functional complexes with different carriers, make new opportunities for the development of advanced smart nanomaterials.

Several studies have identified the potential application of carbon-based stimuli-responsive nanomaterials in disease diagnosis and treatment. Because of the remarkable effect of carbon-based stimuli-responsive nanomaterials in disease treatment, especially in cancer treatment, many researchers are devoted to compounding them with other functional nanomaterials to build novel disease diagnosis and treatment platforms. Zhao et al. [4] prepared stimuli-responsive nanomaterials with integrated cancer diagnostic and therapeutic functions by compounding carbon dots with fluorescent and photoacoustic imaging capabilities as well as photothermal and photodynamic cancer therapeutic functions with gels that have glutathione and pH responsiveness. The composite is stimulated by the tumor microenvironment (TME) and progressively cleaved. During this process, the material is gradually released. Chen et al. [5] prepared carbon-based stimuli-responsive nanomaterials Fe-Ce6-RCDs with the fluorescence imaging function and photodynamic, photothermal, and chemodynamic therapeutic functions by compounding Fe^3+^, a photosensitizer dihydro porphyrin e6 (Ce6) with carbon dots with red light emission. Chen et al. constructed a carbon-based stimuli-responsive nanoplatform, capable of fluorescence imaging-guided cancer therapy under 660 nm laser irradiation to achieve effective treatment of cancer.

**Fig. 1.**
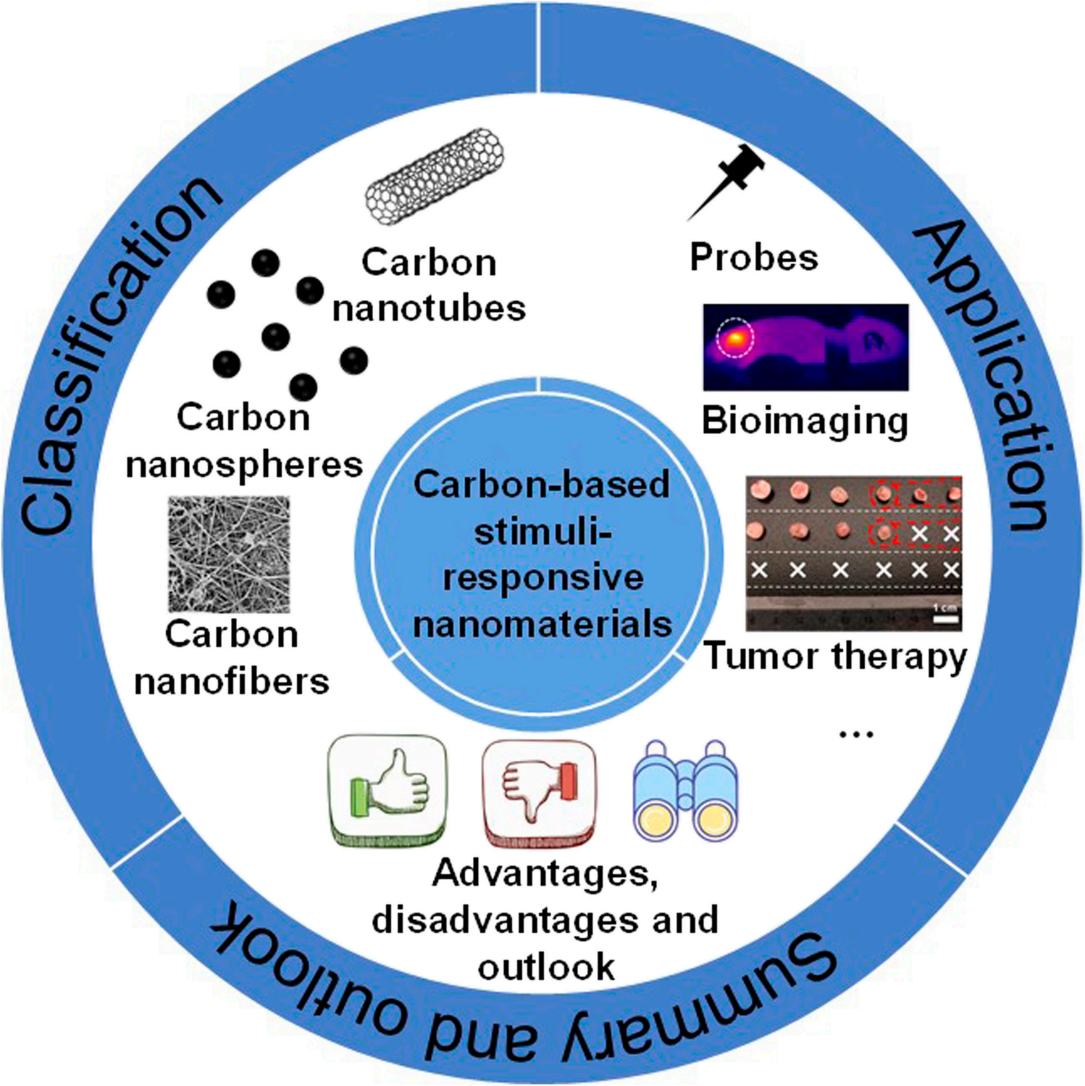
Classification, application, summary, and outlook of carbon-based stimuli-responsive nanomaterials.

The traditional method of classifying carbon-based nanomaterials is mainly based on the microstructure as well as the physical and chemical properties of the materials. Here, we classify carbon-based nanomaterials into 3 main categories: carbon nanotubes, carbon nanospheres, and carbon nanofibers, based on their macroscopic shapes and properties. Then, we discuss the applications of carbon-based stimuli-responsive nanomaterials in probes, bioimaging, tumor therapy, and other fields. Among them, the applications of the material in bioimaging and tumor therapy are focused on. Finally, based on the literature reported so far, we analyze and summarize the advantages and disadvantages of carbon-based stimuli-responsive nanomaterials and provide a future perspective of their application (Fig. 1).

## Classification of Carbon-Based Nanomaterials

With the increasing demand for carbon nanomaterials in industry, the world has witnessed a significant increase in a variety of high-quality new carbon-based nanomaterials and synthesis methods in recent decades [6]. Based on the fact that carbon nanomaterials have great differences in size, shape, composition, etc., there are many ways to classify carbon nanomaterials [7]. In general, researchers are used to dividing carbon nanomaterials into 0-dimensional, 1-dimensional, and 2-dimensional carbon nanomaterials according to their spatial dimension characteristics. In this chapter, we use the traditional classification method to classify carbon nanomaterials into 3 main categories: carbon nanotubes, carbon nanospheres, and carbon nanofibers according to their relevant physical and chemical properties, which is more concise. Carbon nanotubes and carbon nanofibers are also members of the most typical one-dimensional nanomaterial family, while carbon nanospheres, carbon dots, etc., are very typical zero-dimensional nanomaterials. At the same time, several carbon-based stimuli-responsive nanomaterials involved in cutting-edge research at the frontier of science and engineering are listed, and more specific differences are reflected from the perspective of the synthesis and preparation methods of various carbon nanomaterials.

### Carbon nanotubes

In 1991, Ijima [8] successfully discovered carbon nanotubes using a transmission electron microscope and elucidated their structure. Carbon nanotubes are a kind of one-dimensional carbon nanomaterials with a unique hollow tubular shape and are capped by a hemispherical fullerene structure, which is essentially a capped structure formed by curling several layers of graphene. Moreover, nanotubes are mainly generated by the bottom-up method [9]. Since carbon nanotubes were formally discovered and reported, they have been a unique research hotspot in the field of carbon nanomaterials. Current research has not been limited to the exploration of their physical and chemical properties, while the developments of their industrial macro-scale preparation and related application fields have become a focus issue.

There are 3 main ways to synthesize carbon nanotubes: chemical vapor deposition (CVD) method, arc discharge method, and laser ablation method. The properties of the obtained carbon nanotubes depend to a large extent on the selected precursor materials, synthesis methods, and synthesis conditions. Among them, the arc discharge method and the laser ablation method will generate more impurities, yet CVD is regarded as the most economical and productive method [10]. Therefore, the researchers mentioned in this paper use carbon nanotubes prepared by CVD. CVD aims to introduce hydrocarbons or carbon-containing oxides into a high-temperature tubular furnace containing catalysts to form carbon nanotubes after catalytic decomposition. In particular, CVD can synthesize carbon nanotubes at a lower temperature.

Carbon nanotubes can be further divided according to their layers, morphology, and other aspects. In this paper, the most classical layer classification is adopted. Carbon nanotubes can be composed of single-layer or multilayer carbon atomic structures, according to which they can be divided into single-walled carbon nanotubes (SWCNTs) and multiwalled carbon nanotubes (MWCNTs) [11].

#### SWCNTs

In 1993, Ijima and Ichihashi [12] obtained SWCNTs by adding the catalyst to the graphite electrode by the arc discharge method. SWCNTs contain monolayer graphene, whose outer diameter is usually 1 to 2.4 nm, with an average diameter of 1.4 nm, and a length range of 2 to 5 μm [13]. When the diameter exceeds 6 nm, their structure will become unstable. SWCNTs are highly concerned because of their small size, high modulus, and high electrical conductivity [14]. Carbon nanotubes allow multiple molecules to efficiently load along the wall through stacking π–π interactions. At the same time, the related properties can be optimized using a surface chemical modification of their internal and external surfaces [15]. Therefore, SWCNTs have great potential as polymer materials in many fields, such as electronics, machinery, biomedicine, and so on.

**Fig. 2.**
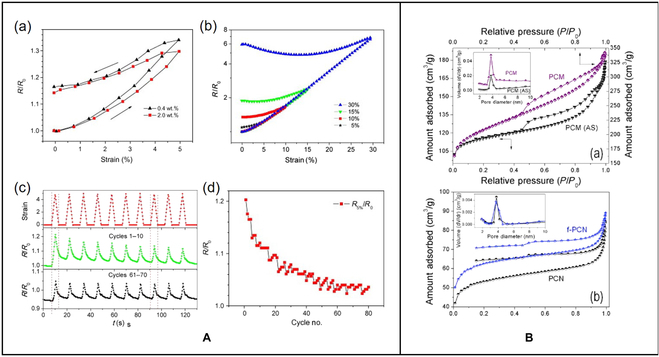
(A) (a) Resistance–strain relationship of CNT/TPU composites with different carbon nanotube loads; the strain rate is as high as 5%. (b) The first cycle of the relative resistance–strain relationship of CNT/TPU composites under different peak strains. (c) Recyclability of CNT composites under cyclic loading. (d) The relative resistance (*R*_5%_/*R*_0_) [20]. Copyright 2013, Elsevier Ltd. (B) (a) Adsorption isotherms and pore size distribution of polycarbonate and polycarbonate. (b) Adsorption isotherms and pore size distribution of polycarbonate and f-polycarbonate [35]. Copyright 2016, Elsevier BV.

The sidewalls of carbon nanotubes are highly hydrophobic and have poor dispersibility in most solvents [16]. To improve the property defects of carbon nanotubes, Liu et al. [17] have been conducting research for years and proposed to covalently combine oxidized SWCNTs with hydrophilic polyethylene glycol (PEG) to achieve functionalization of the sidewall. This kind of SWCNTs can respond to pH stimulation and can be non-covalently combined with many aromatic molecules for further applied research (Fig. 2B).

Shape memory materials can respond to external stimuli such as light and heat, and then quickly restore their original shape. They have attracted much attention due to their low cost, easy processing, and response characteristics [18]. Lee and Yu [19] prepared low-temperature electroactive shape memory materials by blending low-molecular-weight soft-segment polyurethanes with SWCNTs. It is found that SWCNTs have a significant positive effect on the performance of the composite.

#### MWCNTs

MWCNTs are composed of multilayer graphene sheets curled around the central axis. The number of layers is generally in the range of 2 to 50, the distance between layers is about 0.34 nm, and the outer diameter is 2.5 to 100 nm [13]. MWCNTs are also one of the most popular carbon-based nanomaterials with excellent photothermal and mechanical properties. In addition, the production cost of MWCNTs is lower than that of SWCNTs and has higher strength and stiffness. They can be produced in large quantities in the industry, which is of great development value. Zhang et al. [20] developed a CNT/thermoplastic polyurethane (TPU) composite material with a low percolation threshold and significant dispersibility using TPU combined with MWCNTs. It can change its properties such as resistance after stress at different amplitudes (Fig. 2A). You et al. [21] prepared hyperbranched polyamine functionalized multiwalled carbon nanotube gel through a multistep Michael addition reaction. This gel can respond to ultrasonic and rapidly switch between gel and sol in the face of different levels of stimuli. Dang et al. [22] observed the response of MWNTs/poly(vinylidene fluoride) nanocomposites to pressure stimulation. The dielectric constant and electrical conductivity of the composites always decreased after stretching. At the same time, the concentration of MWNTs will affect the sensitivity of the material to tensile stimuli.

### Carbon nanospheres

Carbon nanospheres are a general classification of carbon-based nanomaterials with spherical structures. As one of the most common zero-dimensional carbon nanomaterials, carbon nanospheres even have a longer development history than carbon nanotubes. In 1985, when only 2 carbon allotropes, diamond and graphite, were discovered, Kroto et al. [23] discovered a fullerene composed of 60 carbon atoms: C60. As a result, the research on carbon nanospheres in the field of carbon nanomaterials ushered in an upsurge, followed by the discovery of various carbon nanospheres.

The main synthesis methods of carbon nanospheres are hydrothermal synthesis, template method, emulsion polymerization, and so on [24]. The template method is divided into the hard template method and the soft template method. The hard template method is mainly used for the synthesis of porous carbon nanospheres, which refers to using materials like silica spheres with a rigid framework of the template, depositing carbon precursor on the template by CVD and other methods, and finally removing the template to obtain carbon nanospheres. Xie et al. [25] synthesized carbon nanospheres by hydrothermal synthesis at 180 °C for 10 h. Emulsion polymerization is the most common method for preparing monodisperse polymer carbon spheres, which can be regarded as an improvement of the soft template method. Peng et al. [26] used the emulsion polymerization method and added 1,3,5-trimethylbenzene mediators to prepare nitrogen-doped mesoporous carbon nanospheres with tunable pore size and high uniformity.

According to the shape and structural characteristics of carbon nanospheres, they can be classified into solid carbon nanospheres, hollow carbon nanospheres, porous carbon nanospheres, etc. Porous carbon nanospheres include microporous carbon nanospheres, mesoporous carbon nanospheres, and so on. In this chapter, hollow carbon nanospheres and porous carbon nanospheres with relatively high frequency in the field of stimulus response are briefly introduced.

#### Hollow carbon nanospheres

**Fig. 3.**
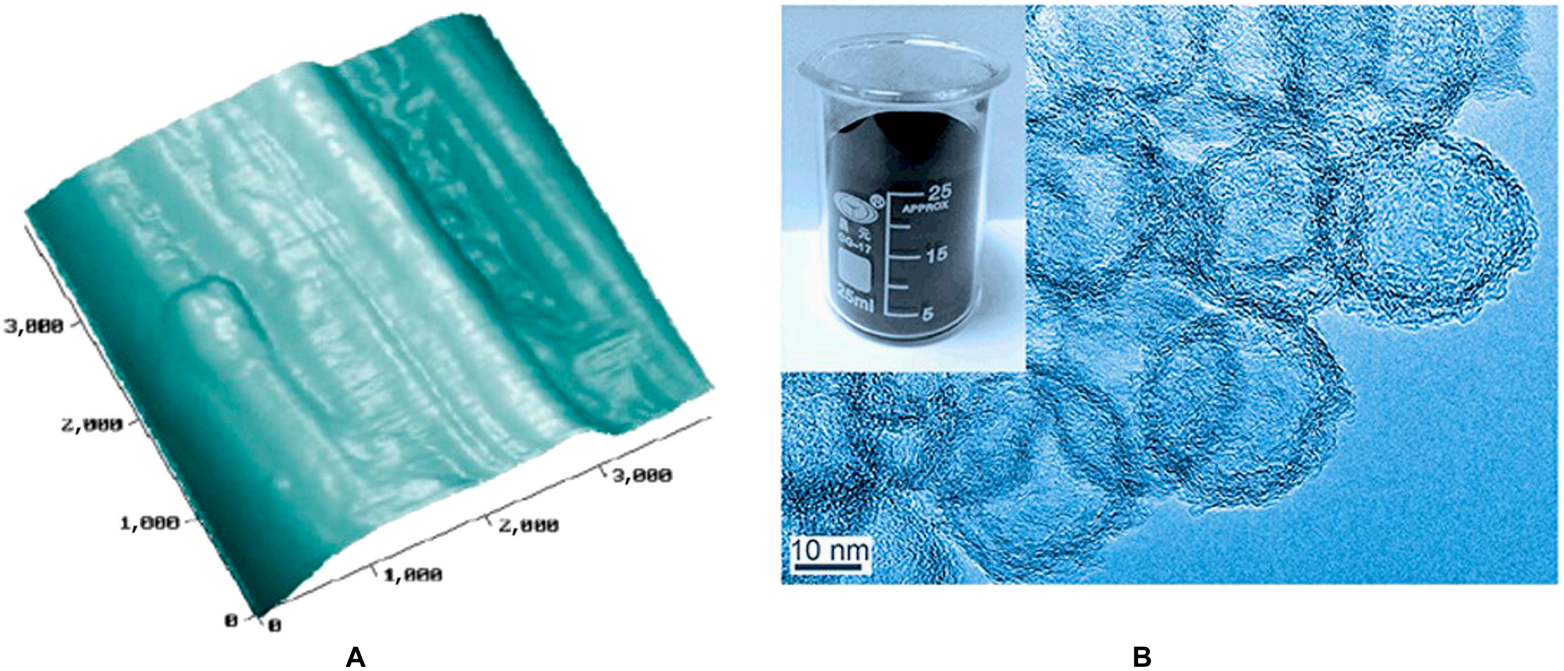
(A) AFM image of the surface of the carbon fibers [39]. Copyright 2010, Elsevier BV. (B) Transmission electron microscope images of hollow carbon nanospheres and digital images of the products [29]. Copyright 2018, Springer Nature.

The hollow carbon nanospheres have low density, strong chemical stability, rich pore structure, and high specific surface area, and the inner diameter of the cavity is about 8 nm and can be loaded with drugs. The hollow structure allows carbon-based materials to excel in many areas such as energy storage [27], since it provides charge storage sites [28] and fast pathways for electron transport [29] (Fig. 3B). Liu et al. [30] surface-modified graphitic hollow nitrided carbon nanospheres with multifunctional polysaccharide hyaluronic acid (HA) to improve their biocompatibility, while generating an enzyme/light dual stimulus-response mechanism. When the complex binds to hyaluronidase, the complex pores open to expose the graphitic hollow carbon nitride nanosphere (GHCN). GHCN then produces reactive oxygen species (ROS) in response to light stimulation. Wang et al. [31] combined TiO_2_ with hollow carbon nanospheres. The formed complex was able to absorb carbon dioxide in response to ultraviolet stimulation. The hollow carbon nanostructure enables light to be scattered multiple times inside the composite material, which greatly improves the light absorption efficiency. The specific surface area of the composite material is extremely large, which effectively improves the adsorption efficiency of carbon dioxide.

As mentioned above, the study of polymer–inorganic hybrid nanomaterials has received increasing attention from researchers. Zhao et al. [32] synthesized 4 different types of carbon nanoparticles by the template method, and then used CD-poly(glycidyl methacrylate) (PGEA) as the coating polymer to make 4 kinds of hybrid nanomaterials. In further experimental verification, they found that among the 4 hybrid materials prepared, the hybrid nanomaterials prepared from rough hollow carbon nanospheres (RHNS) showed the most excellent near-infrared light (NIR) sensitivity, and RHNS has the highest photothermal conversion efficiency. This is largely due to the hollow structure of the RHNS carbon core itself, which makes the hybrid material finally have a rough hollow structure, and then obtain the corresponding stimuli-responsive properties. In summary, hollow carbon nanospheres have a high specific surface area and large pore volume and can be used as effective drug carriers.

#### Porous carbon nanospheres

Non-porous carbon nanospheres perform poorly in chemical loading, while the porosity of porous carbon nanospheres enables them to have outstanding performance. The porous structure makes carbon-based materials have a large number of applications in energy [33], environmental protection, catalyst [34], water treatment, CO_2_ capture, energy conversion, and so on. Kapri et al. [35] prepared porous carbon nanospheres with a diameter of 150 nm using cymbopogon flexuosus as raw material and then functionalized them with chemicals such as doxorubicin (DOX) to obtain pH-responsive properties. Based on porous carbon nanoparticles, there have been studies on the use of nanoparticles to modify them to improve the performance of stimulus response. Zhang et al. [36] encapsulated mesoporous CuS nanoparticles with carbon spheres. The NIR absorption and photothermal conversion of CuS nanoparticles endow them with the response to light, and the heat generated can promote the release of loaded chemicals like DOX.

The stimulus responsiveness of different kinds of carbon nanomaterials is also diverse, which is explored in the related comparative experiments. Zhou et al. [37] prepared mesoporous carbon nanospheres responsive to light through a silica-assisted synthesis strategy. The mesoporous carbon nanospheres have strong absorption efficiency in the UV–Vis–NIR optical region (300 to 1,400 nm). It is found that in the NIR-I and NIR-II, the absorption coefficient of mesoporous carbon nanospheres is 1.5 times higher than that of SWCNTs and graphene, and the photothermal conversion efficiency is higher.

### Carbon nanofibers

Carbon nanofibers generally have a diameter of 10 to 500 nm and a length of about 0.5 to 100 μm. They are fibrous carbon materials between carbon nanotubes and ordinary carbon fibers. The research on carbon fibers can be traced back as early as the 1860s. Carbon nanofibers have a high aspect ratio, high strength, excellent electrical conductivity, strong chemical stability, and anisotropic structure [38]. The unique 3D framework formed by carbon nanofibers makes them flexible instead of brittle, which makes them a new kind of material that has attracted much attention in the industry [39] (Fig. 3A).

Carbon nanofibers are mainly prepared by polymer carbonization, the template method, and the CVD method. Among them, the method of direct carbonization of polymer nanofibers is widely used because of its advantage of being beneficial to industrial large-scale preparation, as the polymer nanofiber materials are usually produced on a large scale using electrospinning technology. In recent years, taking the 2 most studied thermal and pH-responsive polymers, poly(N-isopropylacrylamide) and poly(4-vinyl pyridine), as examples, the polymers with stimulus response are used as nanofiber skeletons [40]. The carbonization method uses responsive electrospinning polymers [41] to obtain carbon nanofibers with good microstructure by rationally controlling the reaction conditions such as heating rate, carbonization temperature, etc.

As mentioned above, the high-volume synthesis of carbon nanofibers usually uses electrospinning technology; therefore, carbon nanofibers are usually classified according to industrial electrospinning synthetic raw materials. In theory, any polymer with a carbon skeleton can be used as a precursor of carbon nanofibers, but in fact, there are not so many kinds of polymers that can produce carbon nanofibers through electrospinning. Among these polymers, PAN has excellent electrospinning property and relatively high carbon yield, and the carbon nanofibers produced at the same time have high chemical stability, mechanical strength, plasticity, etc., which makes it the most commonly used raw material for the production of commercial carbon fibers. In addition, polyacrylate, polyimide, polybenzimidazole, and polyvinylidene fluoride polymer fibers such as phenolic resin and lignin can also be used as precursors of carbon nanofibers. In this section, we divide them into 2 main types: polyacrylonitrile (PAN)-based carbon nanofibers and other carbon nanofibers represented by pitch-based carbon nanofibers.

#### PAN-based carbon nanofibers

At present, PAN is the most important precursor for the synthesis of carbon nanofibers, and its characteristics such as high carbon content and easy carbonization are favored. According to statistics, the precursor in nearly 90% of industrially produced carbon nanofibers is PAN, which is enough to see its irreplaceable position in the synthesis of carbon nanofibers. PAN-based carbon nanofibers also made some achievements in stimulus response.

Lu et al. [42] prepared g-C_3_N_4_/PAN nanofibers by electrospinning, which have visible light stimulus response. The addition of g-C_3_N_4_ with excellent physical properties increases the light absorption capacity of the material, while PAN nanofibers provide a guarantee for the recyclability of the material. Li et al. [43] used PAN carbon nanofibers to prepare carbon nanofiber/silicon composite films with multilayer alternating structures. The properties of PAN carbon nanomaterials such as high electrical conductivity, large specific surface area, and multilevel, multiwave reflection play a key role in the optical/thermal/electrical multiple responses of the carbon nanofiber/silicon composites.

#### Pitch-based carbon nanofibers

Pitch-based carbon nanofibers refer to a type of carbon fibers prepared by electrospinning, carbonization, etc. using materials rich in polycyclic aromatic hydrocarbons such as pitch as raw materials. The precursors of pitch-based carbon fiber are cheap and abundant, and the carbonization yield is also higher than that of PAN-based carbon nanofibers. However, several properties such as the compressive strength of ordinary pitch-based carbon nanofibers are not as good as that of PAN-based carbon nanofibers. Also, the pretreatment process of pitch is cumbersome and expensive; thus, the current cost of high-performance pitch-based carbon nanofibers is higher than that of PAN-based carbon nanofibers. Therefore, their scope of use is relatively limited to the fields of aerospace or military materials. There are relatively few studies in the field of stimulus-response materials using pitch-based carbon nanofibers. However, there are still some cases where they can be used to replace the widely used PAN-based carbon nanofibers.

Carbon nanofibers prepared with PAN as precursors have high costs and relatively low conductivity. To overcome these problems, Jeong and Kim [44] selected a low-cost conventional pitch as a co-recursive agent and added the photo-responsive catalyst ZnO. The properties of the 2 materials are complementary, resulting in a synergistic effect, which, in turn, generates a light-responsive composite. Similarly, Yun et al. [45] used low-cost pitch-based carbon nanofibers and lignin-based carbon nanofibers combined with ZnO to prepare low-cost composites.

## Application of Carbon-Based Stimuli-Responsive Materials

Carbon-based stimuli-responsive materials have been widely studied and applied in many fields, such as probes, bioimaging, and tumor therapy [46]. Carbon-based nanomaterials are used in anti-counterfeiting and optical imaging applications due to their unique optical properties. Conjugation with different targeted detection reagents can increase the sensitivity of carbon-based nanomaterials. Carbon-based nanomaterials can also be used as drug delivery carriers or therapeutic reagents (photothermal, photodynamic, chemotherapy, etc.) for disease treatment.

### Probes

In recent years, carbon-based stimuli-responsive materials have been widely used as fluorescent probes for the detection of various substances and have shown high specificity and sensitivity. It has been widely used in the detection of some typical metal ions in water samples. At the same time, they also show great potential in anions, organic molecules, etc., providing new opportunities for food safety, the chemical industry, and environmental protection.

#### Metal ion probe

Lu et al. [47] synthesized fluorescent carbon nanoparticles for the detection of Pb^2+^ in water. They used grapefruit peel as the carbon source. After hydrothermal treatment, based on the principle of mercury ion-induced fluorescence quenching of carbon nanoparticles, a fluorescent mercury ion probe with a quantum yield (QY) of about 6.9% was prepared. This probe exhibits excellent sensitivity and maintains high selectivity and specificity in the presence of interference from other metal ions. It is worth mentioning that the preparation method is universal. Other fruit peels can also prepare stable fluorescent carbon nanoparticles by this method. Barman and Sadhukhan [48] synthesized blue emission graphitic nitride quantum dots (g-CNQDs) with formamide by the microwave-mediated method, and the QY of g-CNQDs is about 29%. g-CNQDs possess good water solubility and are soluble in most polar solvents. Based on the superquenching of fluorescence, it can be used as a highly sensitive probe for mercury ions in solution, and can also be applied as a detection probe for iodide ions after the formation of g-CNQD-Pb^2+^.

#### Other types of probes

Chen et al. [49] synthesized a fluorescent probe for pesticide residue detection. They designed a composite probe based on blue-emitting nitrogen-doped carbon quantum dots (N-CQDs) and red-emitting copper nanoclusters to construct a novel proportional fluorescence detection method. The probe system is highly selective and sensitive, maintaining good detection function at very low concentrations.

### Bioimaging

**Fig. 4.**
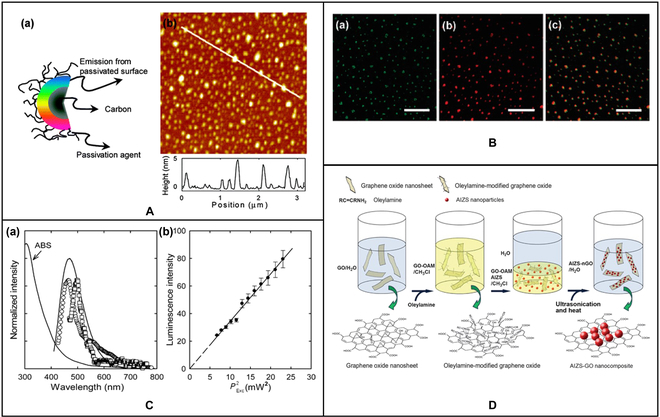
(A) (a) Structure of carbon dot. (b) AFM image of carbon dot. (B) Luminescence image of carbon dot: (a) argon ion laser excitation at 458 nm and (b) femtosecond pulse laser excitation at 800 nm; (c) is a superposition of (a) and (b). (C) (a) The single-photon (□, 458 nm excitation) and 2-photon (○, 800 nm excitation) luminescence spectra of carbon dots. (b) The observed quadratic relationship between the 2-photon emission intensity of carbon dots [50]. Copyright 2007, American Chemical Society. (D) Schematic diagram of the synthesis process of fluorescent AIZS-GO nanocomposites [51]. Copyright 2013, American Chemical Society.

Fluorescence imaging is one of the most widely used imaging techniques in the field of biomedical research. It is of great significance for the identification of lesions and subsequent diagnosis. With the deepening of research on carbon-based stimuli-responsive materials, more and more carbon-based materials have been used for in vivo and in vitro imaging [50] (Fig. 4A), which has become a research hotspot in multidisciplinary fields such as materials, optics, and biomedicine [51] (Fig. 4B).

#### In vitro bioimaging

Jiang et al. [52] used 3 different phenylenediamine isomers to produce carbon dots by the solvothermal method. These carbon dots emit bright, stable red, green, and blue light when excited by a single UV light. At the same time, these carbon dots also have outstanding cell imaging ability and low cytotoxicity, which has potential applications in the field of biological imaging. Liu et al. [53] used dimethyl formamide as the solvent and nitrogen source to prepare N-GQDs by the solvothermal method and conducted in vitro bioimaging studies using human cervical cancer HeLa cells. They found that N-GQDs could label the cell membrane and cytoplasm of HeLa cells without significant infiltration into the nucleus. Coupled with the efficient uptake of N-GQDs by cells, N-GQDs can be used as a kind of 2-photon probe for high-contrast biological imaging.

#### In vivo bioimaging

Compared with in vitro bioimaging, in vivo bioimaging also needs to consider the metabolism of carbon-based materials in vivo while maintaining the low cytotoxicity and excellent imaging capability required by the former. In addition, adverse effects on non-imaging sites should also be considered.

Liu et al. [54] used a one-step hydrothermal synthesis method to produce efficient red luminescent nitrogen-doped carbonized polymer dots (CPDs). Red luminescent CPDs not only retain the original high QY and good biocompatibility but also have outstanding water solubility. When used as fluorescent probes for in vivo bioimaging, most CPDs and their metabolites can be quickly eliminated from the body through urine and can easily cross the blood–brain barrier. This provides a good reference for the imaging and subsequent diagnosis of brain diseases.

### Tumor therapy

#### Photodynamic therapy

Photodynamic therapy (PDT) is a medical technology with rapid research progress, which has made remarkable achievements in the treatment of malignant tumors and various benign diseases. Photosensitizer, light, and oxygen are the 3 basic elements of PDT. The photosensitizer absorbs the energy in the photon to transition to the excited state, and the excited state photosensitizer transfers the energy to oxygen, producing ROS with cytotoxicity [55].

By assembling carbon dots (CDs-Ce6) and Cu^2+^, Sun et al. [56] easily constructed a nanoplatform for TME stimuli-responsive fluorescence imaging and synergistic cancer therapy. They also found that compared with CDs-Ce6, Cu/CC NPs could play a better role in PDT treatment in TME under the same wavelength of laser irradiation. Bai et al. [57] developed carbon dots (S, N-CD) co-doped with sulfur and nitrogen atoms with the redshift absorption effect. The fluorescence properties of the carbon dots are pH sensitive and can be used to detect the tumor modulation process. In addition, S, N-CD nanoparticles can also be used as a PDT/photothermal therapy (PTT) synergistic therapeutic agent, which has no obvious side effects on non-focal major organs, showing good biocompatibility and low cytotoxicity.

#### Photothermal therapy

PTT has developed rapidly in the field of antitumor therapy because of its non-invasive, spatiotemporal controllability and high efficiency. PTT based on nanomaterials has been widely used in clinical treatment and laboratory scientific research [58]. During PTT, laser irradiation is used to enrich tumor sites with photothermal conversion reagents (PTAs) to convert light energy into heat energy. Especially in the near-infrared (NIR) biological window (700 to 1,400 nm), PTT can achieve deep tissue penetration and reduce the thermal effect of tissue, thereby reducing the photodamage to adjacent healthy organs and tissues. Compared with traditional treatment methods, external laser stimulation-mediated nanomaterial-based PTT has the advantages of non-invasiveness, spatiotemporal controllability, good targeting, and low toxic side effects on normal tissues [59]. However, due to the insufficient PTA’s photothermal conversion rate, long-term retention in the body, treatment in the process of tumor recurrence and metastasis, the thermal damage to normal tissue around the tumor, tumor heat resistance, and the existence of such problems as limitations of single therapy, PTT faces enormous challenges in the process of the clinical transformation.

Ge et al. [60] prepared novel carbon dots using the conjugated polymer polythiophene propionic acid (PPA) as a precursor. These carbon dots exhibit up to 38.5% photothermal conversion efficiency under 671 nm laser irradiation and can be used for cancer diagnosis and PTT in living mice.

Qiu et al. [61] synthesized hollow mesoporous carbon nanospheres (HMCNS) with uniform size. The carbon nanospheres have excellent photothermal conversion efficiency and biocompatibility, which can effectively accumulate in tumors. More interestingly, HMCNS can encapsulate drugs and release the drugs under NIR irradiation, which is a powerful assistant for chemotherapy and PPT combined therapy. This study also provides a new idea for the material design of carbon nanomaterials in cancer treatment.

### Other fields

Anti-counterfeiting labels based on luminescent nanoparticles have great potential in anti-counterfeiting applications. With the in-depth exploration of carbon-based stimuli-responsive materials, fluorescent materials have become one of the important directions of anti-counterfeiting research. It is very important to improve the stability and simplify the preparation process while making good use of the fluorescence properties. Liu et al. [62] prepared nitrogen-doped carbon dots (N-CDs) after the hydrothermal method using citric acid (CA) and tris (hydroxymethyl)methyl aminomethane. The carbon dots have a high QY of 75% and maintain excellent light stability over a wide temperature range. The composite material of N-CDs can be used for article packaging and can provide an advanced anti-counterfeiting function for luxury and valuable personal items. Kalytchuk et al. [63] synthesized fluorescent CDs with good water solubility through a simple hydrothermal route using CA and ethylenediamine. By changing the reaction temperature from 200 °C to 220 °C, 2 lifetimes of fluorescent CDs were synthesized: fast (CDs-f) and slow (CDs-s). They have the same emission color but different luminescence kinetics. CDs exhibit a longer fluorescence lifetime; thus, the prepared CD inks can be used to form anti-counterfeiting labels through fluorescence lifetime coding.

Carbon-based stimulated materials have attracted extensive attention in the fields of fluorescence sensing and optoelectronic devices because of their unique luminescence properties. Sensors and light-emitting devices based on CBN have been widely studied and applied in the actual production process, which has become another exploration point of the integration of materials and optoelectronics.

Liu et al. [64] studied a graphene photodetector with high broadband and high responsiveness, which can reach the detection level of the most advanced infrared photodetector operating at low temperatures. The detector consists of 2 layers of graphene sandwiched in a thin tunnel barrier, with hot electrons and holes separated into opposing graphene layers by selective quantum tunneling, thus minimizing hot carrier recombination. The deep exploration of the mechanism of a hot carrier tunnel in a graphene bilayer heterostructure has greatly improved the sensitivity of light detection, which provides a feasible way for subsequent research.

Briscoe et al. [65] applied CQDs to the solar cells. CQDs produced by the top-down method can be used as sensitizers for ZnO nanorods, with lower cost compared with traditional methods. However, the light-harvesting efficiency of CQD is limited, which greatly restricts the efficiency of solar cells. The use of thicker CQD coating or size control may be used in future studies.

## Summary and Outlook

### Advantages

**Fig. 5.**
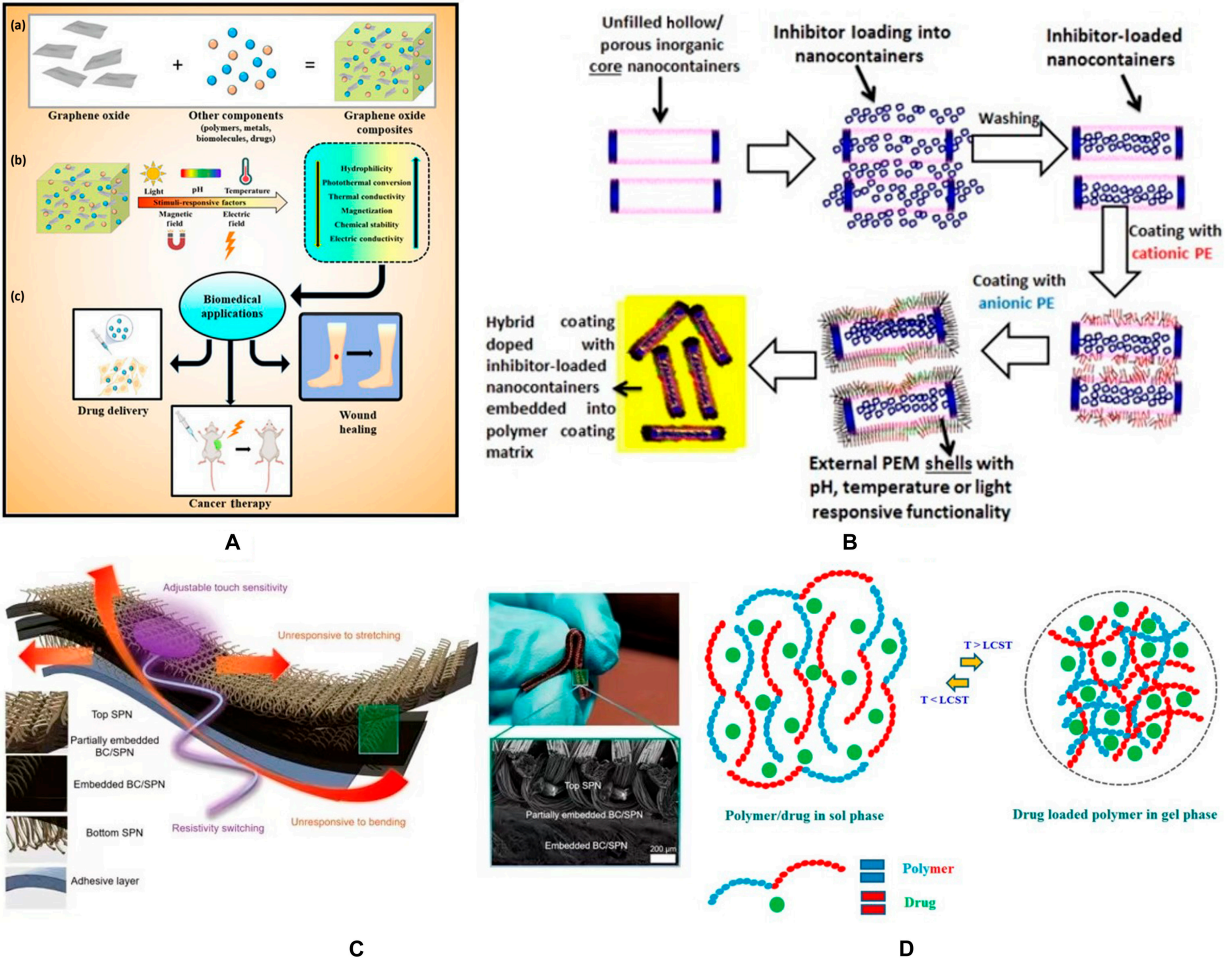
(A) Schematic diagram of composite materials formed by cross-linking graphene oxide with various polymers, biomolecules, drugs, and metals. (a) Composite formation by cross-linking graphene oxide with various polymers, biomolecules, drugs, and metals; (b) effect of stimuli factors such as pH, light, thermal, magnetic, and electric fields on graphene oxide-based materials; (c) application for wound healing, drug delivery, and cancer therapy [66]. Copyright 2021, *MDPI*. (B) Diagram illustration of the effects of pH, light, heat, magnetic, electric field, and other stimulating factors on graphene oxide-based materials [67]. Copyright American Chemical Society. (C) Schematic diagram of the preparation of hybrid nanocontainers consisting of loaded inhibitor inorganic core nanotubes coated with responsive PEM shells [72]. Copyright 2016, Elsevier BV. (D) Schematic diagram of the thermal response behavior of a stimuli-responsive polymer hydrogel drug delivery system [76]. Copyright 2019, MDPI.

Carbon-based stimuli-responsive materials have excellent physical and chemical properties. After analyzing the current research progress, it was concluded that carbon-based stimuli-responsive materials have great potential for applications [66] (Fig. 5A). Due to their lattice structure, they not only have excellent thermal, electrical, and photosensitive properties but also have other good physical properties such as high mechanical strength and so on [67] (Fig. 5B). In some studies, the physical and chemical properties of the materials can be easily modified by chemical functionalization, which makes the materials more suitable for practical application. In this section, these advantages are analyzed and summarized.

Carbon-based stimuli-responsive materials have good biocompatibility, high water solubility, low toxicity [68], and easy surface functionalization. Based on their super-high surface area, excellent physical and chemical properties, and antibacterial ability, carbon-based stimuli-responsive materials have great clinical application potential in wound healing, drug delivery, and cancer treatment [69].

At the same time, their strong light absorption rate and lipophilicity can increase cell permeability [70], thus realizing the integration of diagnosis and treatment. At present, there are 2 common synthesis methods of carbon-based stimuli-responsive materials: the top-down method, which reduces or decomposes large-scale materials into nanoscale elements; the bottom-up method, using small carbon precursors to synthesize materials with large atomic mass, such as graphene [71]. Synthetic carbon-based stimulation response materials have stronger water solubility, and larger surface area, and are reported to be better carriers, which can load drugs, cell-targeted ligands, nucleic acids, and protein more effectively [72] (Fig. 5C).

### Disadvantages

Although carbon-based stimuli-responsive materials are increasingly used for in vivo therapy and in vitro perception, some physical and chemical defects, toxicity, and lack of clinical research are still problems that need to be faced. In this section, combined with the main application fields of carbon-based stimuli-responsive materials, some of their defects in current research are summarized.

The toxicity of carbon-based stimuli-responsive materials is a major clinical difficulty. In terms of chemical composition, C is the fundamental structural unit of carbon-based stimuli-responsive materials, which have very low toxicity. When clinical trials are conducted, they will accumulate in cells and tissues, and there are some potential dangers to life systems [73]. At present, carbon-based stimuli-responsive materials are usually surface-functionalized. In the process of functionalization, physical and chemical stimuli are used for surface modification, and there are some heavy metal materials [74] in the composition of the materials. The addition of heavy metal materials will bring certain toxicity. The mechanism of toxicity is currently being accepted by researchers, which is the oxidative damage of carbon-based stimuli-responsive materials to cells, which affects the membrane passage and even penetrates cell membranes, thus interfering with the normal physiological activities of organisms [75].

**Fig. 6.**
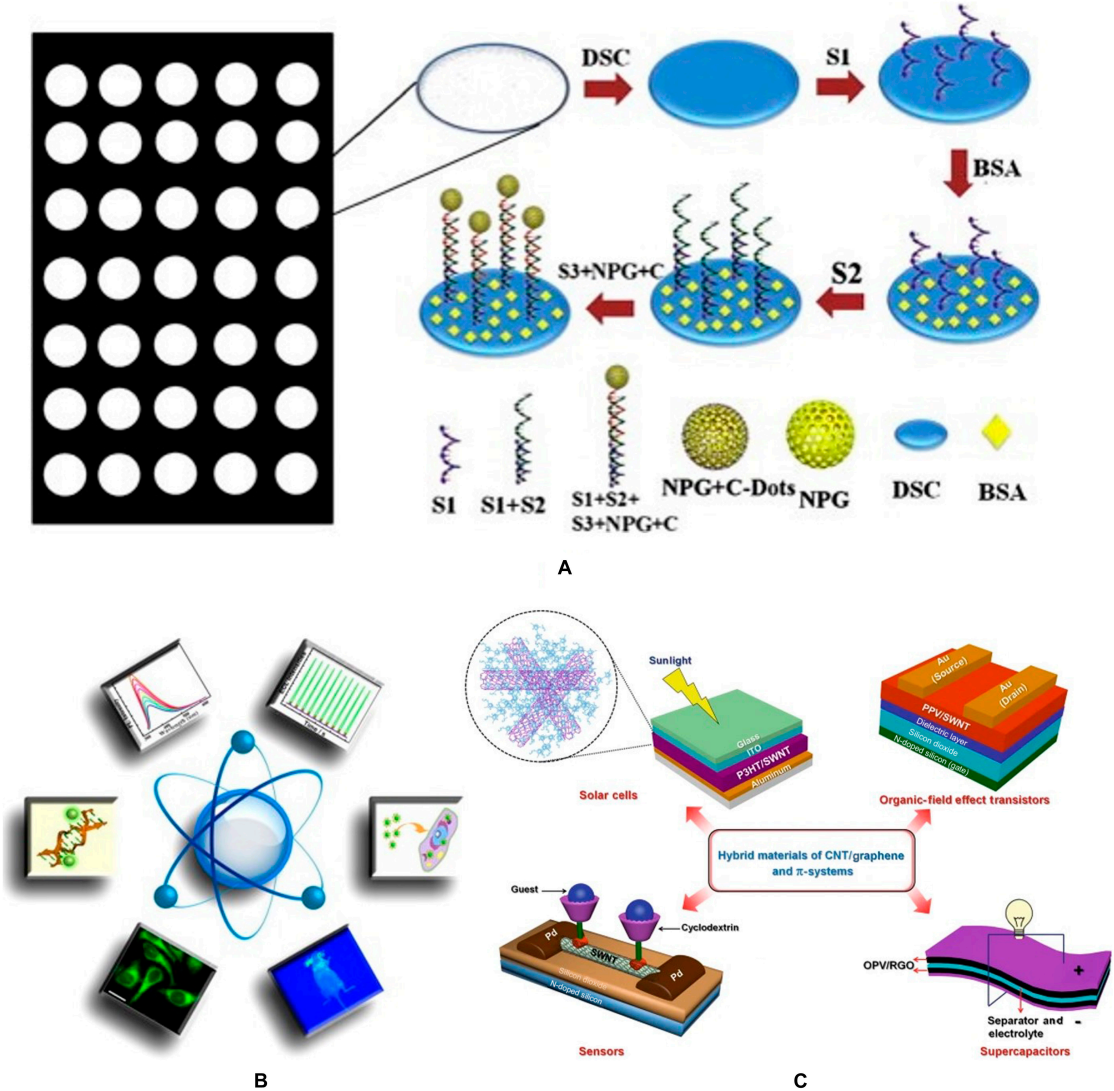
(A) Diagram for the preparation of carbon dot-based bio-DNA detection sensor. (B) Legend of CNT/graphene and π-system hybrid materials in some representative applications [77]. Copyright 2015, Elsevier BV. (C) Schematic diagram of the biological applications of carbon dot [80]. Copyright 2018, Nature Publishing Group.

In the past 10 years, the safety of carbon-based stimuli-responsive materials is a research hotspot [76] (Fig. 5D). It is generally accepted that carbon-based stimulation response materials have inherent insolubility, which leads to their poor biocompatibility, so they may have asbestos-like toxicity. Drug delivery systems use this property to deliver drugs [77]. At the same time, carbon-based stimuli-responsive materials are sensitive to the degradation of enzymes; thus, we should analyze them concretely and evaluate their biocompatibility [78,79] (Fig. 6A and B). There are potential and promising diagnostic and therapeutic applications in cancer therapy, but there are not enough key clinical experiments for clinical cancer therapy [80] (Fig. 6C). Carbon-based stimuli-responsive materials have made new progress in the field of cancer therapy. At the same time, the lack of a more accurate clinical treatment stage is another major problem faced by carbon-based stimuli-responsive materials. After accurate practice, we should be more forward-looking and fully consider the long-term impact, cost-effectiveness, and large-scale production of the preparation, to provide large-scale effective treatment and realize personalized diagnosis and treatment.

The challenging problems faced by carbon-based stimuli-responsive materials are more than biocompatibility and cytotoxicity. At present, there are some challenging problems, such as uncontrollable aggregation, immunogenicity, possible side effects, and adverse reactions. Solving these problems is very important to the clinical application of carbon-based stimuli-responsive materials. Therefore, to overcome these challenges, Animal models and long-term monitoring should be implemented.

### Future perspective

Carbon-based stimuli-responsive materials are promising. Their discovery and application will change the prospect of modern science, technology, and engineering. Carbon-based stimuli-responsive materials not only have excellent physical and chemical properties, but also can be combined with other polymers for functionalization, and become excellent carriers for drug delivery and cancer treatment. At present, the safety of carbon-based stimuli-responsive materials is unknown because of the lack of key evidence in medical clinical experiments. However, many studies have shown that carbon-based stimuli-responsive materials can effectively become materials that play an important role in the biomedical field through hybridization. To better reduce the controversy of carbon-based stimuli-responsive materials and increase credibility, it is necessary to conduct in-depth research on toxicology, pathology, and biodynamics. In the future, researchers are expected to develop new synthesis methods or create new composites to improve the cure rate of malignant tumors and improve human life.

## Data Availability

The raw/processed data required to reproduce these findings cannot be shared at this time as the data also form part of an ongoing study.
